# Supplementary material for: Late Miocene Tarim desert wetting linked with eccentricity minimum and East Asian monsoon weakening
Source: Nat Commun. 2022 Jul 8;13:3977. doi: 10.1038/s41467-022-31577-w (PMC9270403; doi:10.1038/s41467-022-31577-w)
Supplement: Supplementary file 1 — Supplementary Information [file 41467_2022_31577_MOESM1_ESM.pdf]

Supplementary Information

Late Miocene Tarim desert wetting linked with eccentricity minimum and East Asian monsoon weakening

Nie et al.

Supplementary figures

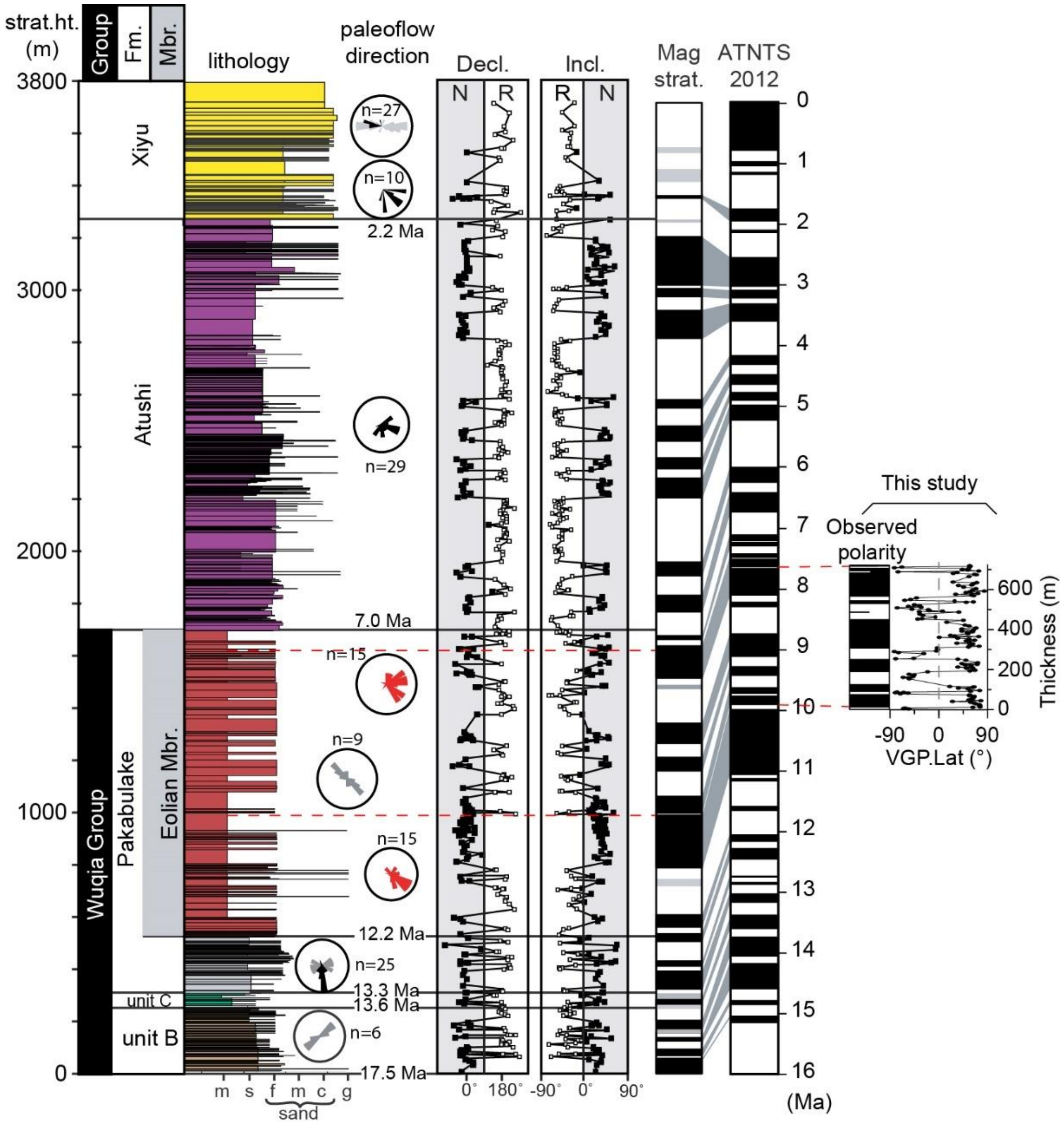

Supplementary Fig. 1 Paleomagnetic dating comparison between this study and a previous study**.** The two red dashed lines show how the sampled interval in this study (right) corresponds to the stratigraphy in Heermance et al. (2018)[1].

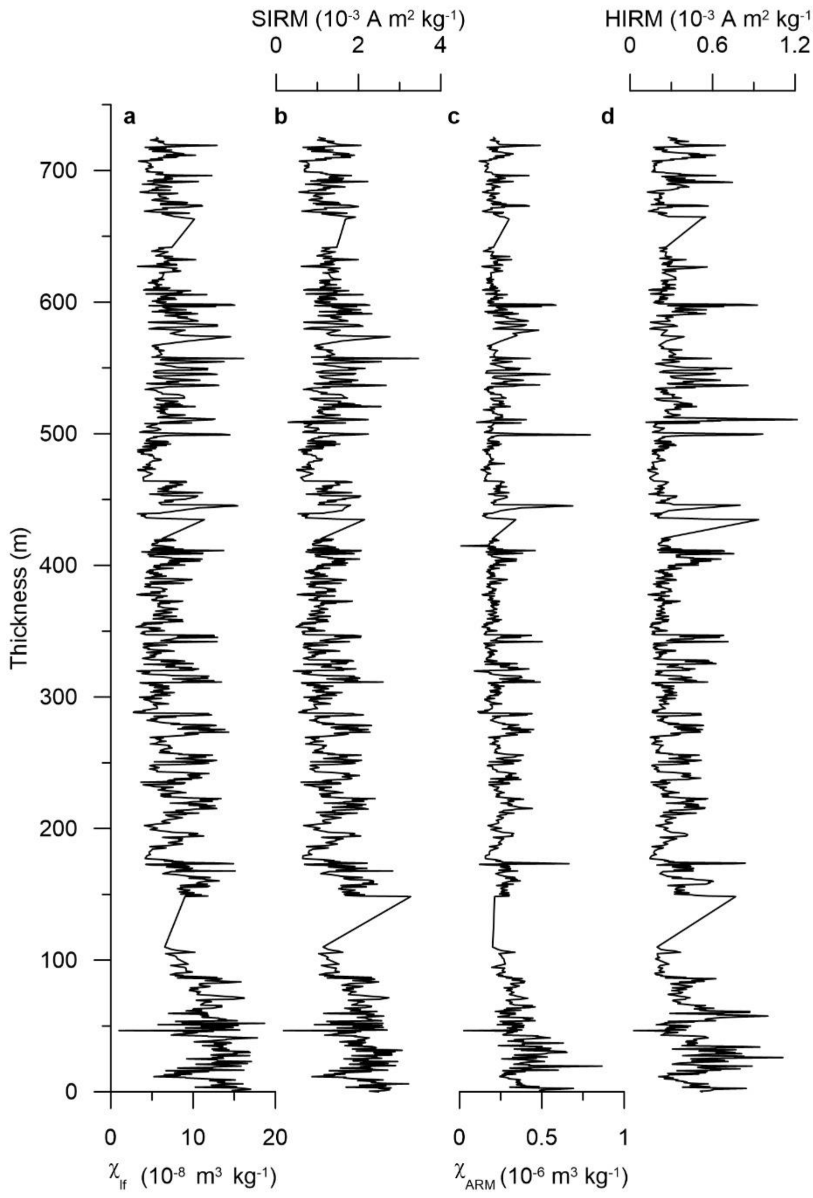

Supplementary Fig. 2 Magnetic parameter records from the studied section**. a,** Low-frequency magnetic susceptibility. **b,** Saturated isothermal remanent magnetization (SIRM). **c,** Susceptibility of anhysteretic remanent magnetization (χ_{ARM}). **d,** Hard isothermal remanent magnetization (HIRM).

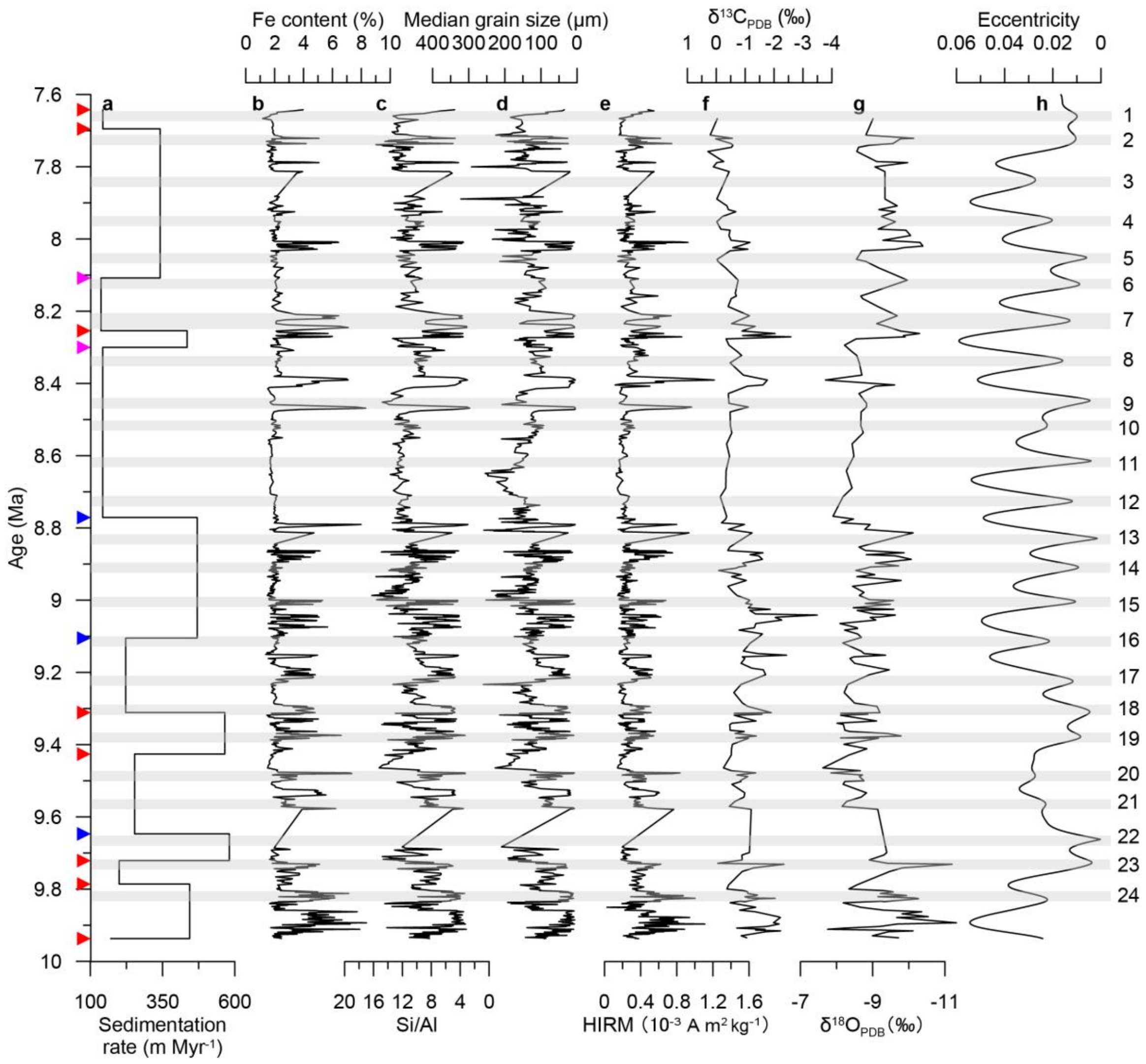

Supplementary Fig. 3 Sedimentation accumulation rate and paleoenvironmental proxy data from the studied section. **a**, Sedimentation rate. **b**, Fe content. **c**, Si/Al. **d**, Median grain size. **e**, Hard isothermal remanent magnetization (HIRM). **f**, $\delta^{13}C_{PDB}$. **g**, $\delta^{18}O_{PDB}$. **h**, Eccentricity[2]. Numbers on the right indicate eccentricity lows from top to bottom. Gray bars correspond to eccentricity lows. Triangles mark geomagnetic reversals recorded in this section, and color regimes are the same as in Fig. 2.

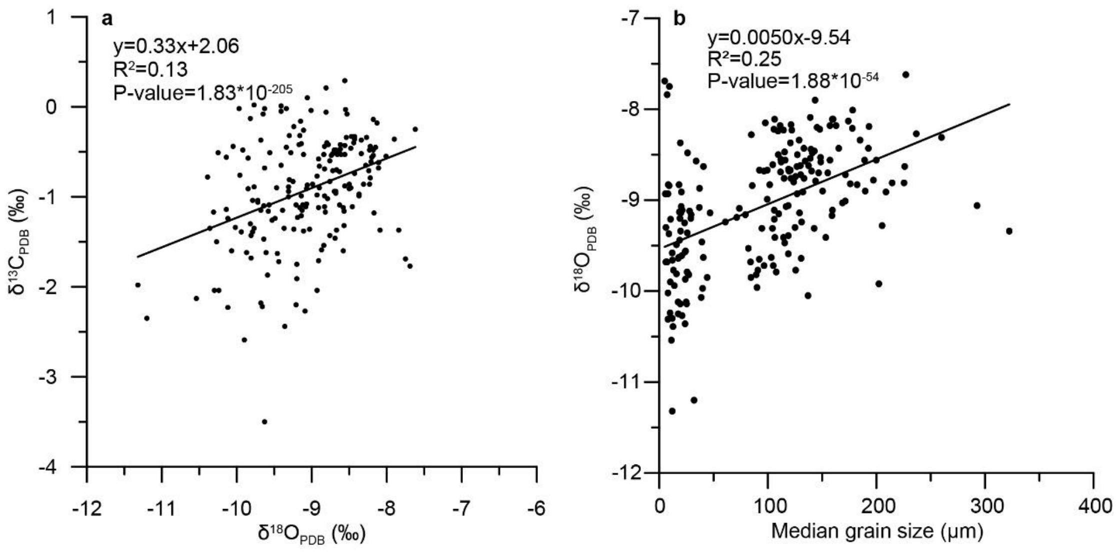

Supplementary Fig. 4 Correlation between $\delta^{13}C_{PDB}$ and $\delta^{18}O_{PDB}$ (**a**), and between median grain size and $\delta^{18}O$ (**b**) in the studied section.

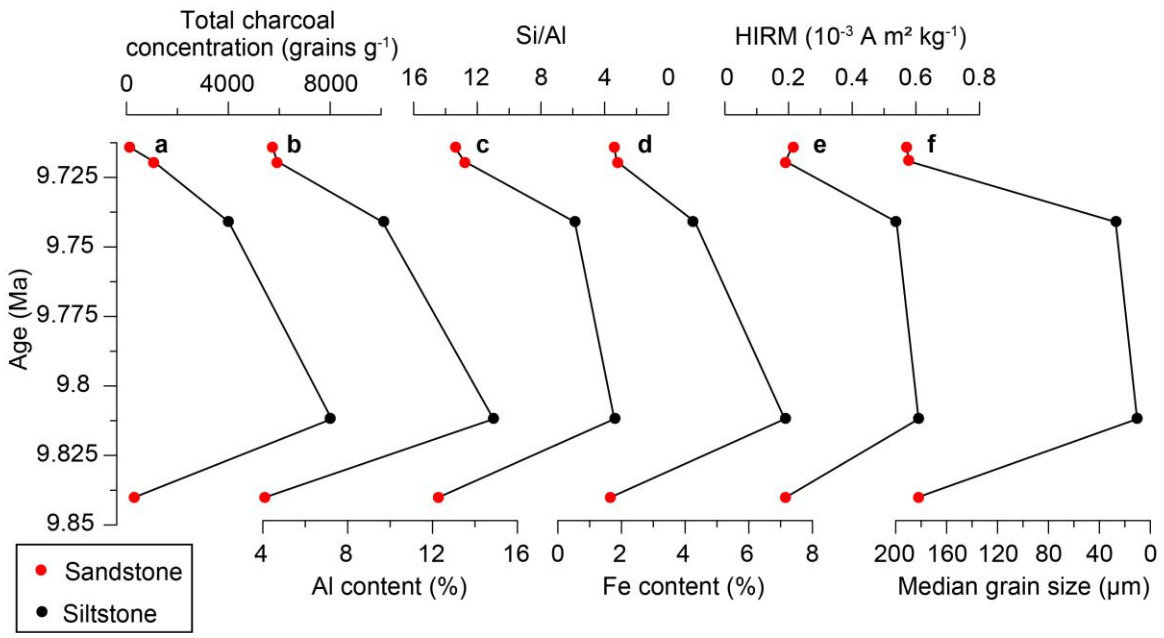

Supplementary Fig. 5 Paleoenvironmental records of the representative samples from the eolian strata (red dots) and lacustrine/fluvial overbank strata (black dots) of the studied section. **a**, Total charcoal concentration. **b**, Al content. **c**, Si/Al. **d**, Fe content. **e**, Hard isothermal remanent magnetization (HIRM). **f**, Median grain size.

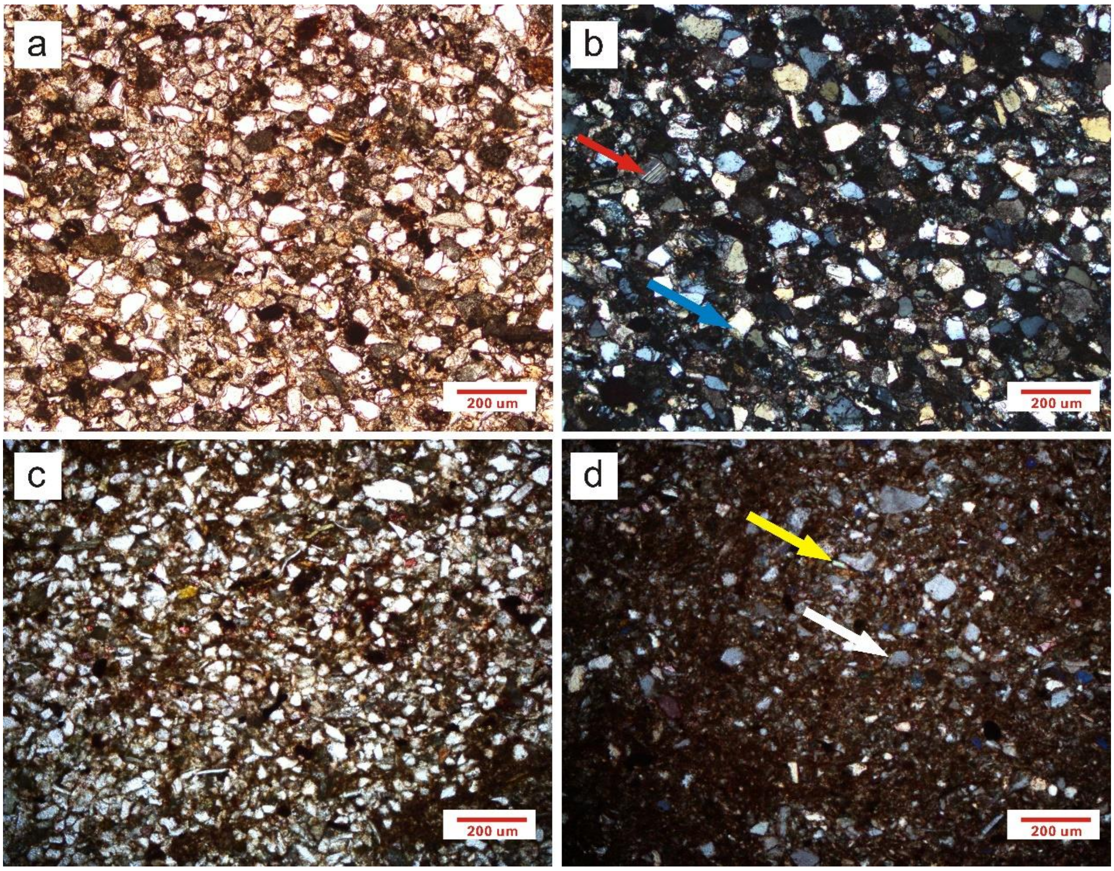

Supplementary Fig. 6 Photomicrographs of two representative samples (sandstone at 557.2 m and siltstone at 86 m) under plane polars (**a, c**) and crossed polars (**b, d**). Sample at 557.2 m is quartz sandstone with carbonate and ferruginous cement. The detrital composition contains >80% quartz, 15% feldspar, few rock debris, and mica. The detrital quartz is indicated by blue arrow and the detrital feldspar is indicated by red arrow in **b**. Sample at 86 m is sandy siltstone with carbonate cement and pelitic texture. The pelitic texture contains about 70% microcrystalline clay minerals, 30% quartz, and few micas. The quartz is indicated by white arrow and mica is indicated by yellow arrow. Detrital carbonate fragments are not observed in the thin sections of these two samples.

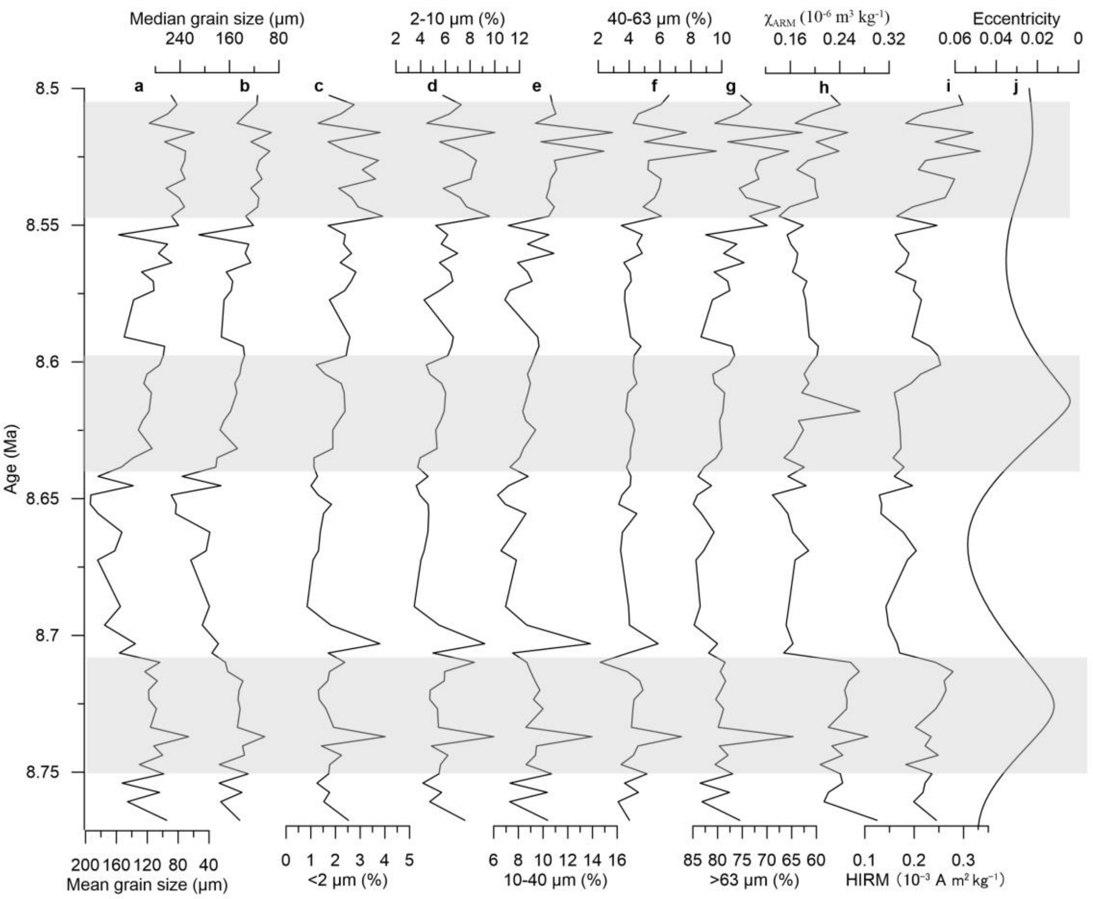

Supplementary Fig. 7 Paleoenvironmental data over 8.8-8.5 Ma and comparison with eccentricity. **a**, Mean grain size. **b**, Median grain size. **c**, Percentage of less than 2 μm grains. **d**, Percentage of 2 to 10 μm grains. **e**, Percentage of 10 to 40 μm grains. **f**, Percentage of 40 to 63 μm grains. **g**, Percentage of more than 63 μm grains. **h**, Susceptibility of anhysteretic remanent magnetization (χ_{ARM}). **i**, Hard isothermal remanent magnetization (HIRM). **j**, Eccentricity[2]. Three eccentricity lows (gray shades) correspond to decreased grain size and increased magnetic parameter values.

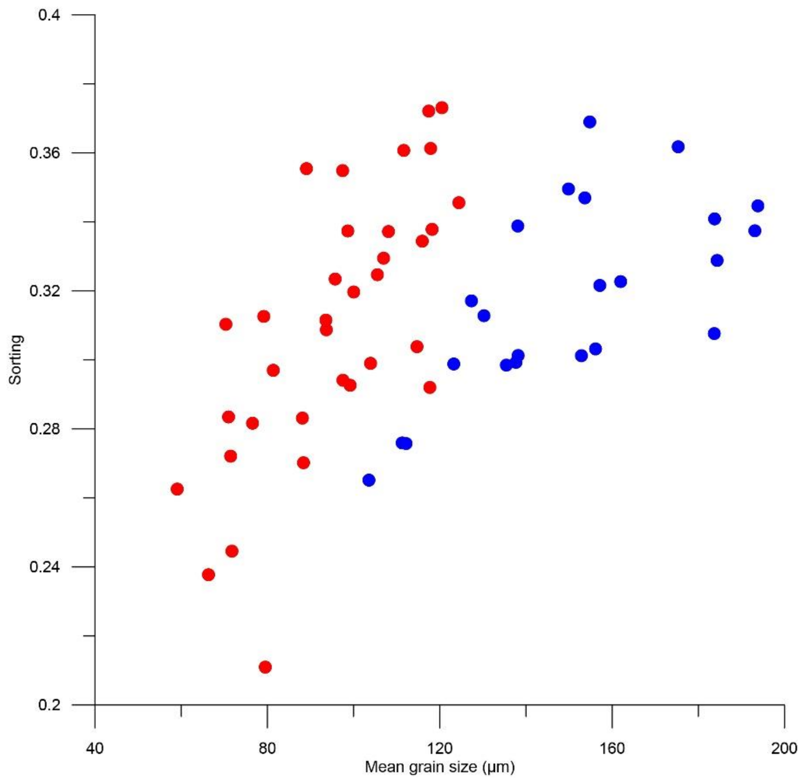

Supplementary Fig. 8 Sorting versus mean grain size for the interval 8.8-8.5 Ma. Red and blue dots correspond to samples aligning with low (highlighted using three gray bars) and high eccentricity intervals in Supplementary Fig. 7, respectively.

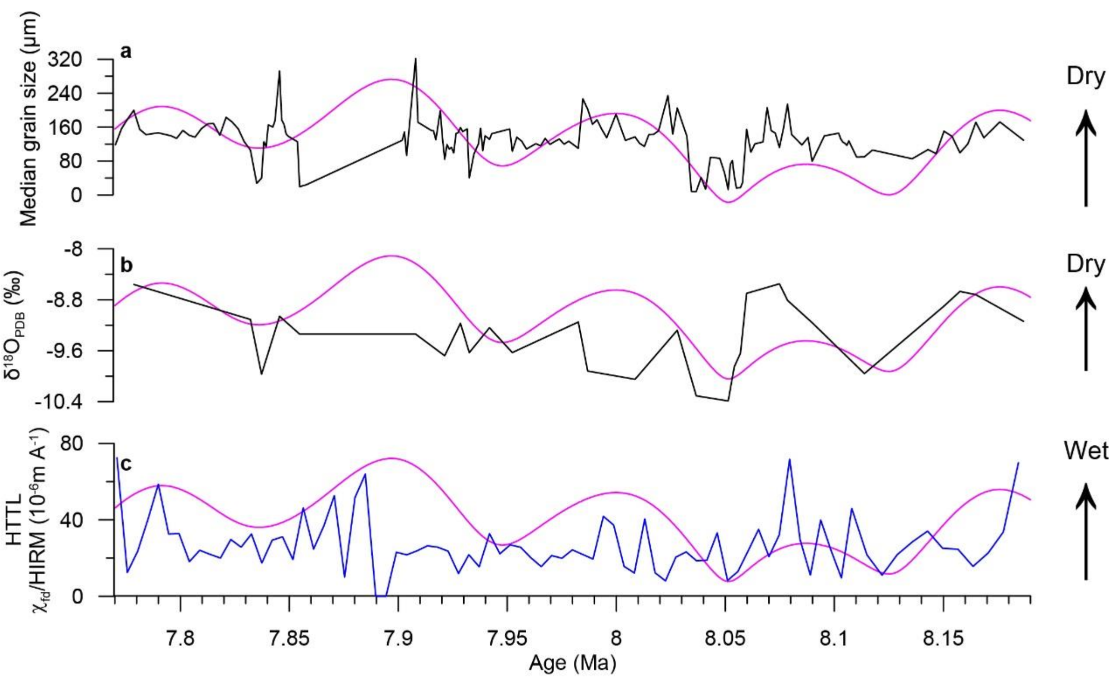

Supplementary Fig. 9 Environmental wet-dry variation comparison for a 400-kyr window between the eastern Qaidam Basin (HTTL section, blue) and the Tarim Basin (this study, black). **a**, Median grain size of the study section. **b**, $\delta^{18}O_{PDB}$ of the study section. **c**, χ_{fd}/HIRM in the HTTL section, Qaidam Basin[3]. Purple curve shows relative variations of eccentricity for comparison, with peaks representing larger eccentricity. Late Miocene Qaidam records are interpreted as reflecting East Asian summer monsoon variations[3].

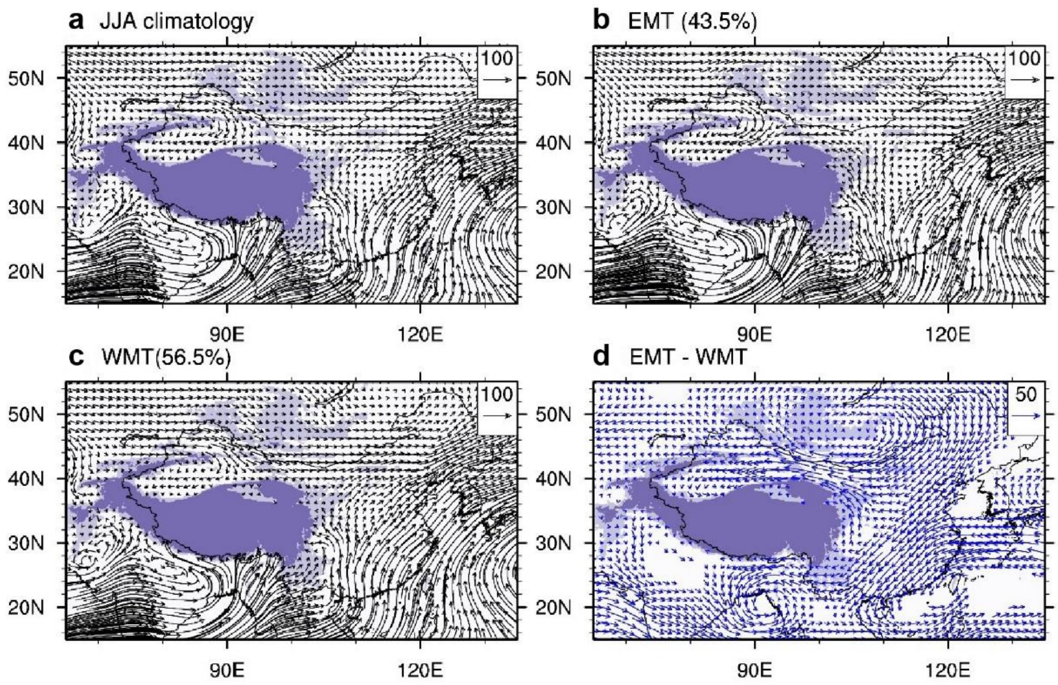

Supplementary Fig. 10 Fluxes of water vaper transport between 1958 and 2019. **a**, Mean fluxes of water vapor (kg·m^{-1}·s^{-1}) in the lower troposphere (surface to 700 hPa) in summer between 1958 and 2019. JJA: June, July and August. **b**, Mean fluxes of water vapor (kg·m^{-1}·s^{-1}) in the lower troposphere during the days with easterly moisture transport (EMT) along the northern periphery of the Tibetan Plateau in summer. Number in the parenthesis indicates the percentage of the EMT events in summer. **c**, Mean fluxes of water vapor (kg·m^{-1}·s^{-1}) in the lower troposphere during the days with westerly moisture transport (WMT) along the northern periphery of the Tibetan Plateau in summer. Number in the parenthesis indicates the percentage of the WMT events in summer. **d**, Comparison of water vapor fluxes between the EMT and WMT events. Blue vectors indicate differences that are significant at the 0.05 confidence level. Areas above 1500 m and 3000 m are shaded by light and dark purples, respectively. The climate data is from the Japanese 55-year Reanalysis (JRA-55)[4].

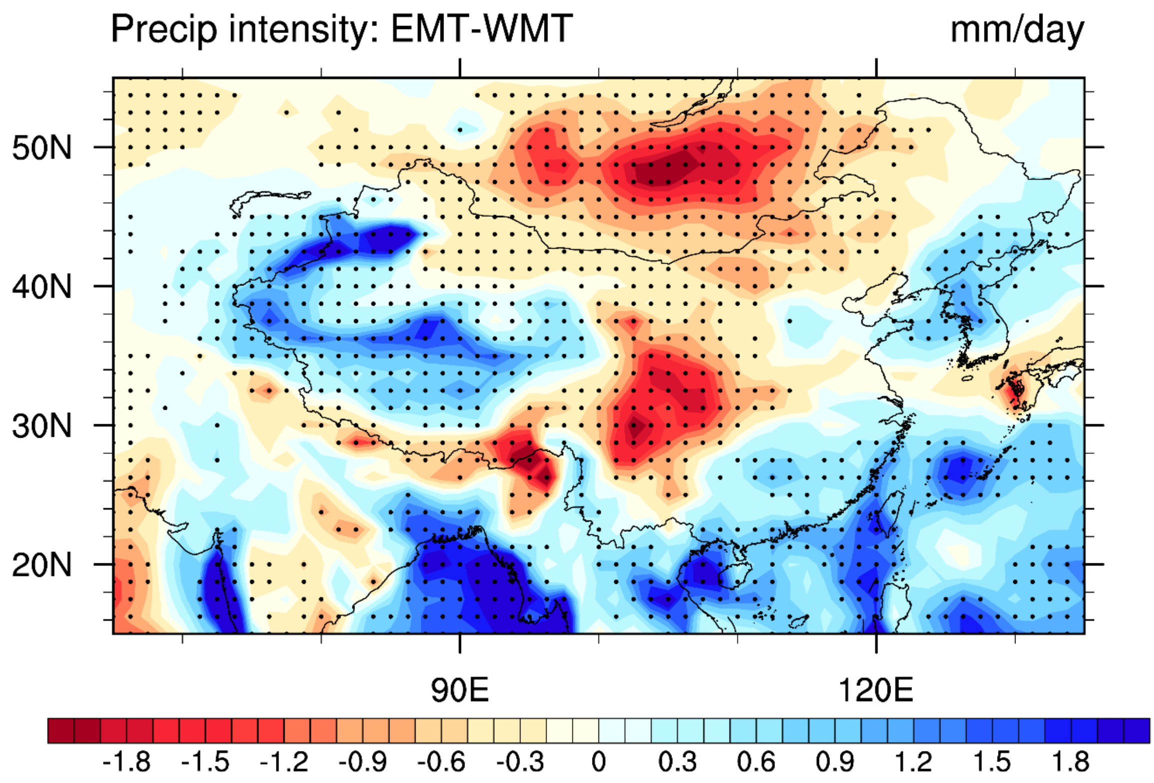

Supplementary Fig. 11 Comparison of mean precipitation intensity (mm/day) between the easterly moisture transport (EMT) and westerly moisture transport (WMT) events. Dotted regions indicate differences that are significant at the 95% confidence level. The climate data are from the Japanese 55-year Reanalysis (JRA-55)[4].

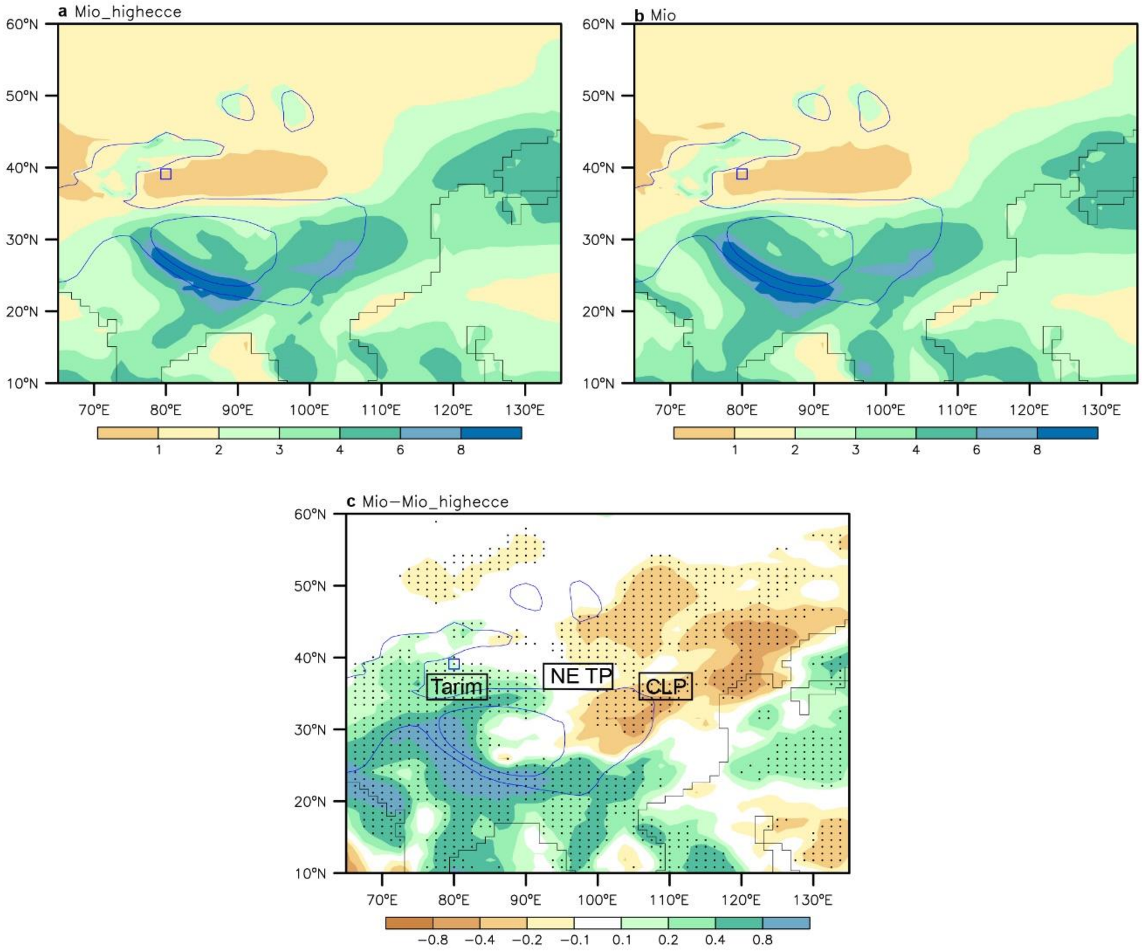

Supplementary Fig. 12 Simulated annual precipitation (shaded, mm day^{-1}). **a**, Results using 10 Ma background boundary conditions and 0.05 eccentricity. **b**, Results using 10 Ma background boundary conditions and 0.01672 eccentricity. **c**, Precipitation differences when increasing eccentricity from 0.01672 (year 1950) to 0.05. Dotted regions indicate differences that are significant at the 90% confidence level. Blue square indicates location of the study site.

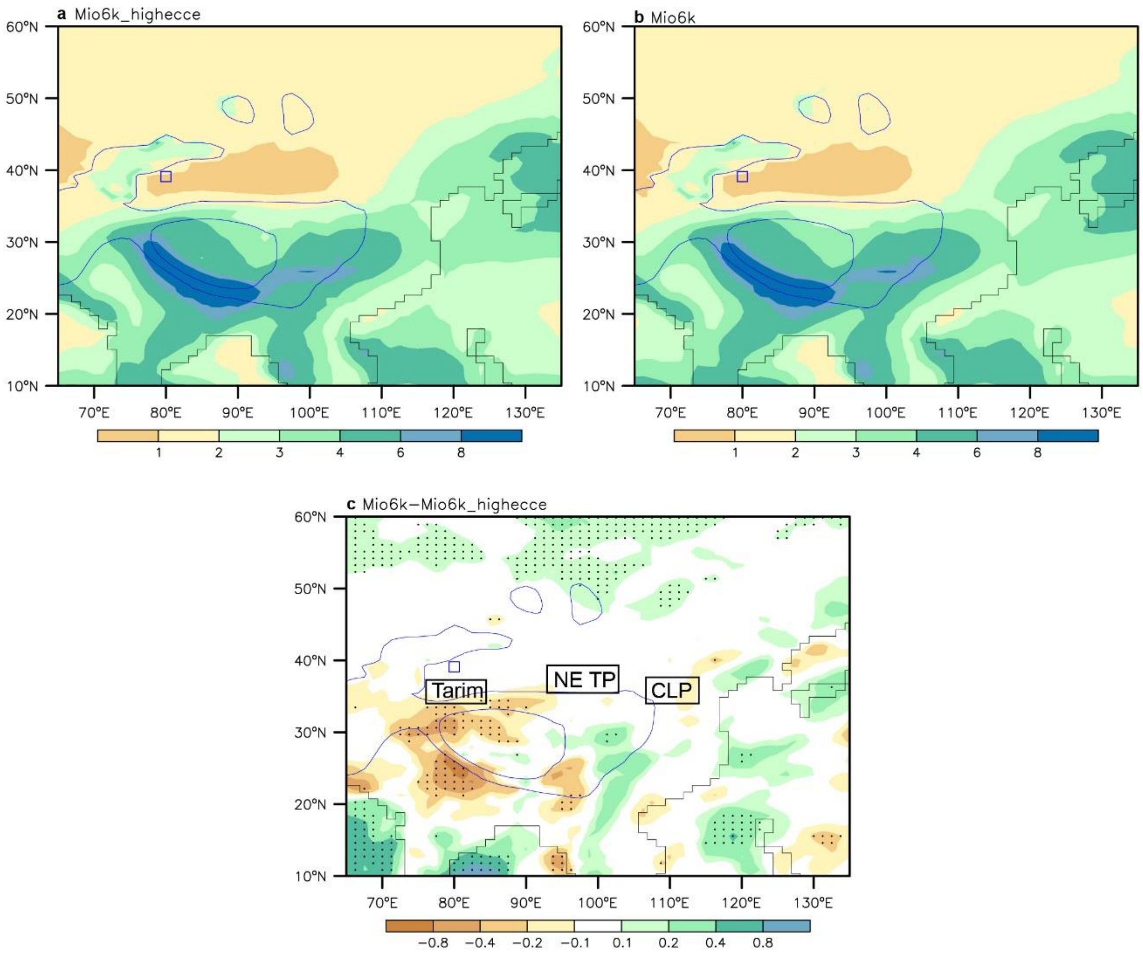

Supplementary Fig. 13 Simulated annual precipitation (shaded, mm day^{-1}) when using 6 ka precession**.** **a**, Results using 10 Ma background boundary conditions, 0.05 eccentricity and 6ka precession. **b**, Results using 10 Ma background boundary conditions, 0.01672 eccentricity and 6ka precession. **c**, Precipitation differences when increasing eccentricity from 0.01672 (year 1950) to 0.05 when using 6k precession. Dotted regions indicate differences that are significant at the 90% confidence level.

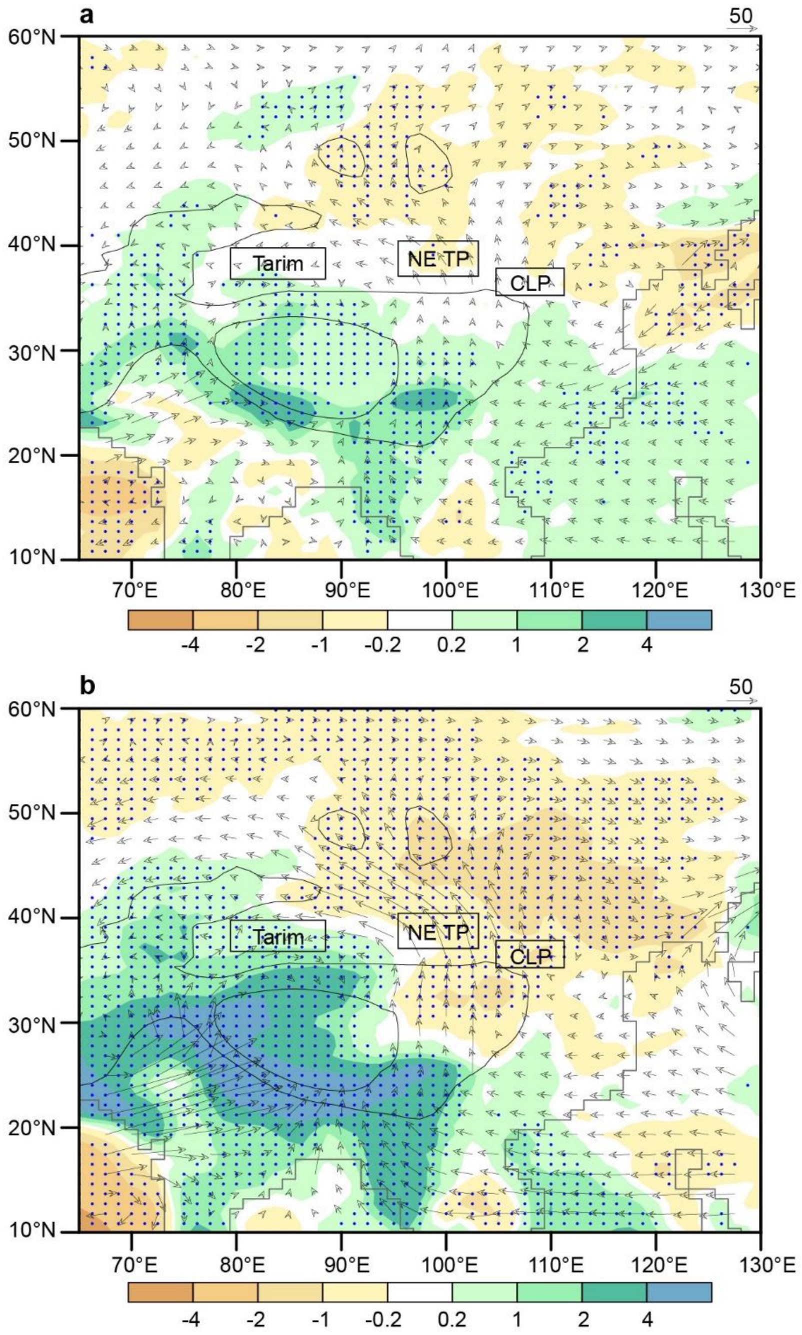

Supplementary Fig. 14 Simulated late Miocene summer precipitation (shaded, mm day^{-1}) differences between 6 ka and 0 ka precessional setting (6ka-0ka) under low (**a**) and high (**b**) eccentricity. The precipitation in Supplementary Fig. 14 is similar to that in Fig. 3c. The experiment settings are outlined in Supplementary Table 2. Eccentricity settings are the same as in Fig. 3: Low eccentricity = 0.01672 (year 1950); high

eccentricity = 0.05. Dotted regions indicate differences that are significant at the 90% confidence level. Squares indicate location of the Tarim Basin, NE Tibetan Plateau (NE TP), and the Chinese Loess Plateau (CLP).

Supplementary Tables

Supplementary Table 1. Geomagnetic reversal ages[5] used in this study, and duration of each reversal in meter and time.

Reversals (Ma)	Reversal duration in meter (m)	Reversal duration in time (kyr)
7.642	3	10
7.695	4	13
8.108	11	36
8.254	4	13
8.3	12	39
8.771	24	78
9.105	23	75
9.311	2	7
9.426	4	13
9.647	38.3	125
9.721	4	13
9.786	5	16
9.937	4	13

Supplementary Table 2. Experimental design.

Experiment	Boundary conditions
Mio	~10 Ma paleogeography and SSTs from NorESM-L experiment; Antarctic ice sheet; Idealized vegetation (broadleaf evergreen temperate shrubs); CO_2 = 350 ppmv; CH_4 = 760 ppbv; N_2O = 270 ppbv; Solar constant = 1365 W m^{-2}; Modern orbital parameters (1950).
Mio_highecco	Similar to Mio, except changing the eccentricity from 0.01672 to 0.05.
Mio6k	Similar to Mio, except using the precession at 6 ka

Supplementary References

1. Heermance, R. V. et al. Erg deposition and development of the ancestral Taklimakan Desert (western China) between 12.2 and 7.0 Ma. *Geology* **46**, 919-922 (2018).
2. Laskar, J. et al. A long-term numerical solution for the insolation quantities of the Earth. *Astron. Astrophys.* **428**, 261-285 (2004).
3. Nie, J. S. et al. Dominant 100,000-year precipitation cyclicity in a late Miocene lake from northeast Tibet. *Sci. Adv.* **3**, e1600762 (2017).
4. Kobayashi, S. et al., The JRA-55 Reanalysis: General Specifications and Basic Characteristics. *J. Meteor. Soc. Japan* **93**, 5-48 (2015).
5. Ogg, J. G. in *The Geologic Time Scale 2012* (eds Gradstein, F. M., Ogg, J. G., Schmitz, M. D. & Ogg, G. M.) 85-113 (Elsevier press, 2012).
